# Alcohol-Induced Cardiomyopathy Presenting With a Ventricular Arrhythmia Storm Causing Cardiac Arrest

**DOI:** 10.7759/cureus.80411

**Published:** 2025-03-11

**Authors:** Esosa U Ukponmwan, Sandeep Banga, George Abela

**Affiliations:** 1 Internal Medicine, Michigan State University-Sparrow Hospital, Lansing, USA; 2 Cardiology, University of Michigan Health-Sparrow Hospital, Lansing, USA; 3 Cardiology, Michigan State University, East Lansing, USA

**Keywords:** alcohol, alcohol use disorder (aud), cardiac arrest, cardiomyopathy, echocardiogram, intubation, transthoracic echocardiogram, ventricular arrhythmia

## Abstract

Alcohol has both protective and deleterious effects on the heart. Alcohol consumed in small quantities can have cardiovascular protective effects. However, consumption of excessive amounts of alcohol has been associated with electrolyte imbalances, neurologic disorders like Wernicke's encephalopathy and Korsakoff syndrome, cardiomyopathies, cardiac arrhythmias and sudden cardiac death. We present and discuss the diagnosis and management of a 26-year-old male patient with a history of alcohol use disorder who presented to our center with alcohol-induced cardiomyopathy causing a ventricular arrhythmia storm and cardiac arrest.

This case highlights the approach to cardiopulmonary resuscitation, describes the diagnosis using electrocardiogram, echocardiogram, and coronary angiogram, medical management of this patient by the Intensivist, and interventions to improve clinical outcome, assist with alcohol cessation, and medical strategies to prevent future occurrences of cardiac events and alcohol related co-morbidities. It is important to recognize that in patients with no other risk factors for cardiac disease, prompt recognition and management, including cessation of alcohol consumption, has been associated with good outcomes and prognosis.

## Introduction

Alcohol is a chemical constituent of alcoholic beverages like beer, wine, cider and spirits. It is the intoxicating substance found in alcoholic beverages. It is also called ethanol or ethyl alcohol. Alcohol has been shown to have both protective and deleterious effects on the heart [1,2]. Studies have shown that white and red wine consumed in small quantities can have cardiovascular protective effects [1-3]. However, consumption of excessive amounts of alcohol, particularly spirits with high percentage alcohol content, has been associated with electrolyte imbalances and cardiotoxic effects causing alcohol cardiomyopathy, cardiac arrhythmias including atrial fibrillation, ventricular arrhythmias, and rarely sudden cardiac death [1-3].

We present and discuss the diagnosis and management of a young male patient who presented to the emergency room of our medical center via the emergency medical services (EMSs) with recurrent cardiac arrests due to a ventricular arrhythmia storm as a result of severe dilated cardiomyopathy caused by excessive alcohol consumption.

## Case presentation

A 26-year-old male with a history of seizure disorder, major depressive disorder, essential hypertension, and a five-year history of alcohol use disorder with consumption of one pint of vodka daily (equivalent to 17 standard drinks) and recent admission for severe alcohol withdrawal presented to our medical center by the EMS.

Our patient was found down in his home by his partner at about 9 am. When EMS arrived about five minutes later, he was found to be in pulseless electrical activity, cardiopulmonary resuscitation (CPR) was immediately started. Patient's cardiac rhythm transitioned to ventricular tachycardia. Patient received three rounds of CPR during which he received epinephrine and was defibrillated three times before return of spontaneous circulation was achieved (ROSC) within six minutes.

**Figure 1:**
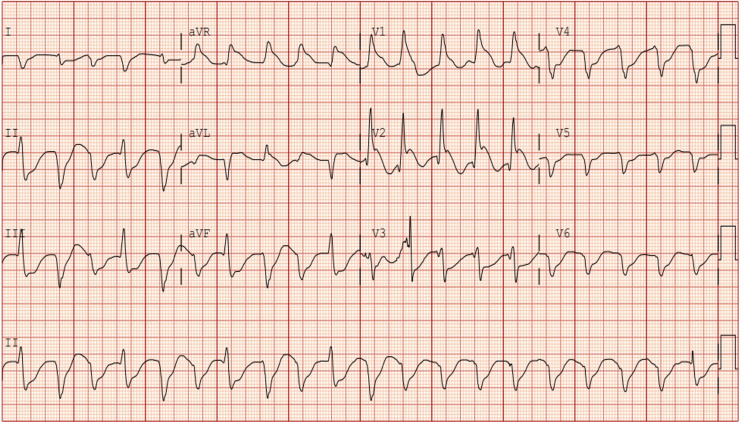
Electrocardiogram at presentation showing wide complex tachycardia with premature ventricular complexes. Heart rate 121 beats per minute.

On arrival to the hospital, the patient became pulseless again, with rhythm notable for ventricular fibrillation. CPR was started, with ROSC achieved within five minutes. He had six total episodes of cardiac arrest requiring advanced cardiac life support protocol with his rhythm alternating between Torsades-de-pointes, pulseless electrical activity, ventricular fibrillation and ventricular tachycardia. He received epinephrine and lidocaine during resuscitation. He was intermittently defibrillated with a total down time of about 45 minutes, before achieving ROSC, he was given an amiodarone bolus and was started on epinephrine and lidocaine drip. The electrocardiogram (EKG) done at presentation showed wide complex tachycardia (Figure 1). 

He was intubated and transferred to the intensive care unit. In the intensive care unit, physical examination was notable for an unconscious male, with a Glasgow Coma score of 3/15 (eye opening 1, verbal response 1, motor movement 1) and myoclonic jerks, and rales in the right lower lung. Otherwise, his physical examination was unremarkable. 

Alcohol screen was negative; however, the patient was reported to have consumed a pint of vodka a day before. He was also recently admitted two weeks earlier for alcohol withdrawal. Urine drug screen was positive for benzodiazepines, barbiturates and cannabinoids.

Significant abnormal laboratory results notable at presentation are displayed in Table 1 with normal reference ranges. Hypokalemia, though mild, has been associated with cardiac arrhythmias. Magnesium was within normal limits; however, hypomagnesemia has been associated with cardiac arrhythmias. Our patient had an elevation in high sensitivity troponin, likely due to non-ischemic cardiomyopathy, reflected by myocardial strain due to alcoholic cardiomyopathy and cardiac arrest with resuscitation. The complete blood count and other laboratory results were within normal limits. Patient had a complete infectious work-up including blood cultures and comprehensive viral panel, which were all negative.

**Table 1:** Abnormal laboratory results on initial presentation.

Parameters	Results	Reference range
Potassium	3.1mEq/L	3.5 -5.0mEq/L
Magnesium	2.3 mg/dL	1.8 -2.6mg/dL
Random blood glucose	222mg/dL	70 to 140mg/dL
B-type natriuretic peptide	157pg/mL	<100pg/mL
High sensitivity troponin at presentation	65ng/L	<14ng/L
High sensitivity troponin 2 hrs. after presentation	30,254ng/L	<14ng/L

Chest x-ray showed right-sided pneumonia; CT scan of the brain and CT angiogram showed no hemorrhage of acute findings. A point of care ultrasound showed global hypokinesis, severely reduced left ventricular ejection fraction of 10% and reduced right ventricular function.

**Figure 2:**
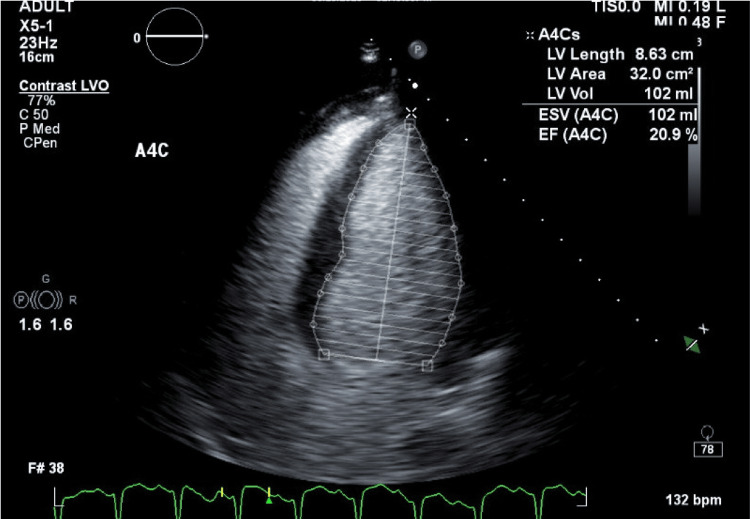
Echocardiogram at presentation showing left ventricular ejection fraction of 20%.

A transthoracic echocardiogram with Simpson's ejection fraction (Figure 2) showed “left ventricular systolic function to be severely reduced with ejection fraction of 20% with global hypokinesis. Grade III diastolic dysfunction with elevated left atrial pressure. Right ventricle size normal with reduced function.” The echocardiogram findings with severe left ventricular dysfunction are consistent with alcohol-induced cardiomyopathy.

**Figure 3:**
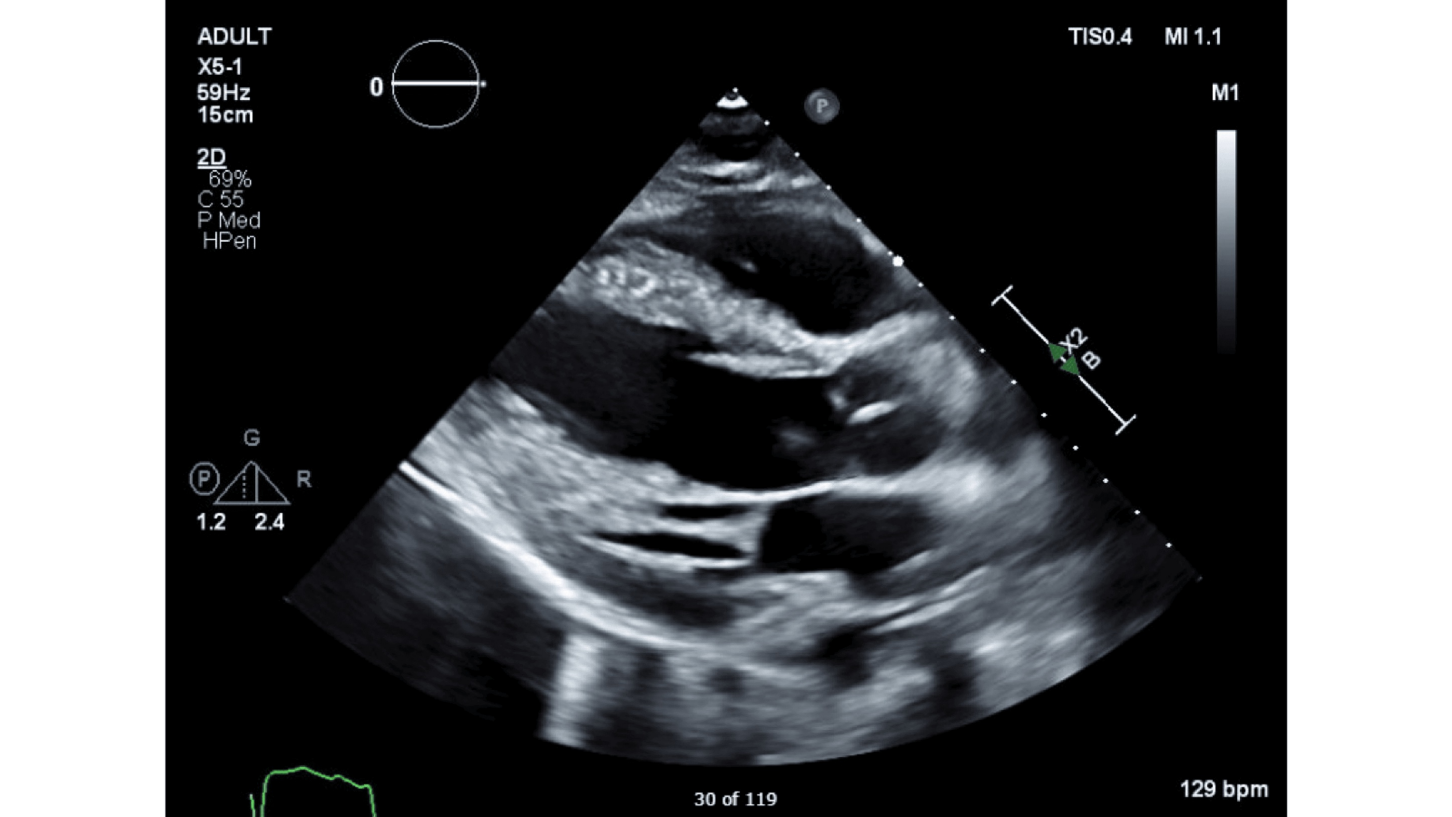
Parasternal long axis view of transthoracic echocardiogram done at presentation showing left ventricular ejection fraction of 20%.

The parasternal long axis view of the transthoracic echocardiogram done at presentation is shown in Figure 3. 

The patient was started on targeted temperature management with a goal temperature 36 degrees Celsius for 24 hours. He was also started on norepinephrine, heparin, fentanyl, and propofol drips. He received levetiracetam for seizures and antibiotics for pneumonia.

All electrolytes were optimized including hypokalemia which was corrected. He was adequately fluid resuscitated. A continuous electroencephalogram (EEG) done showed no consistent focal slowing or interhemispheric asymmetry, no interictal epileptiform discharges or electrographic seizures and was reported as a normal continuous EEG.

Patient made remarkable improvement, with improvement in mentation after 48 hours. By day 2 of admission, sedation was weaned down, and the patient had a spontaneous breathing trial, but failed due to tachycardia and tachypnea. He self-extubated later that day and was monitored closely afterward. His mentation, oxygen saturation and vitals remained stable on close monitoring in the Intensive care unit.

On day 3 of admission, a coronary angiogram was done due to elevated high sensitivity troponin and cardiac arrest at presentation, coronary angiogram showed normal coronary arteries, which ruled out ischemic cardiomyopathy, supporting a diagnosis of non-ischemic alcohol-induced cardiomyopathy. He was started on goal directed medical therapy with Carvedilol, Sacubitril-Valsartan and Spironolactone. He was given a cardiac life-vest which is a wearable cardioverter defibrillator while on admission and eventually had an implantable cardioverter defibrillator (ICD) placed before discharge. He had an ICD placed, so in the event of another ventricular arrhythmia event, the device will deliver a defibrillator cardioversion shock and prevent sudden cardiac death. He was counseled to avoid alcohol, given resources and medical management to assist with alcohol cessation. Patient made a full recovery and was discharged on day 7 to follow up with the cardiologist.

**Figure 4:**
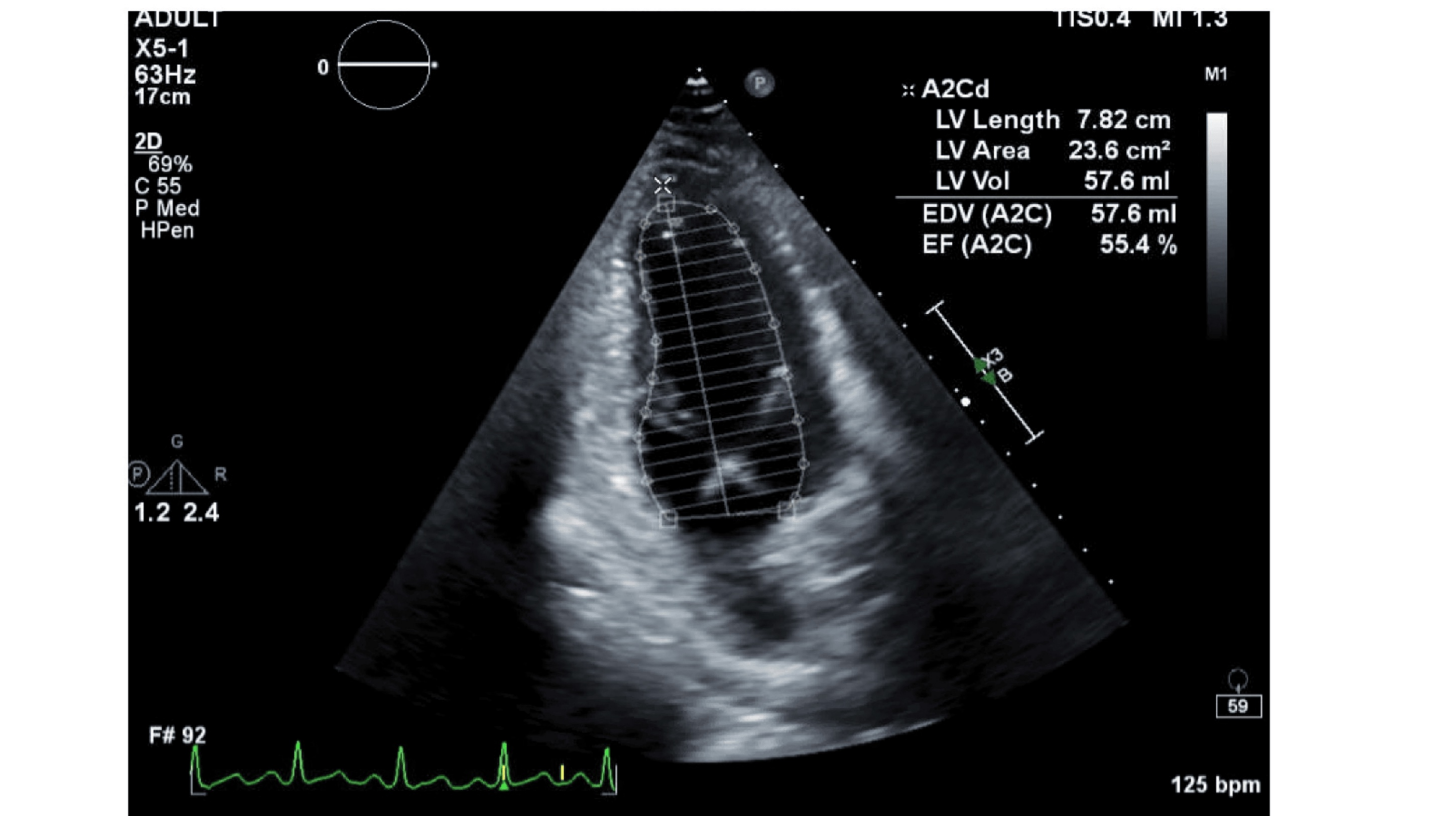
Follow-up transthoracic echocardiogram showing normal left ventricular wall motion and ejection fraction 55% to 60%.

At follow up four weeks after, transthoracic echocardiogram showed improved left ventricular ejection fraction of 55% to 60%. Patient endorsed he had quit alcohol and returned to his normal daily routine. He denied any clinical symptoms and was grateful to the medical team for the care he had received. Figure 4 shows the follow up echocardiogram with ejection fraction 55% to 60%.

**Figure 5:**
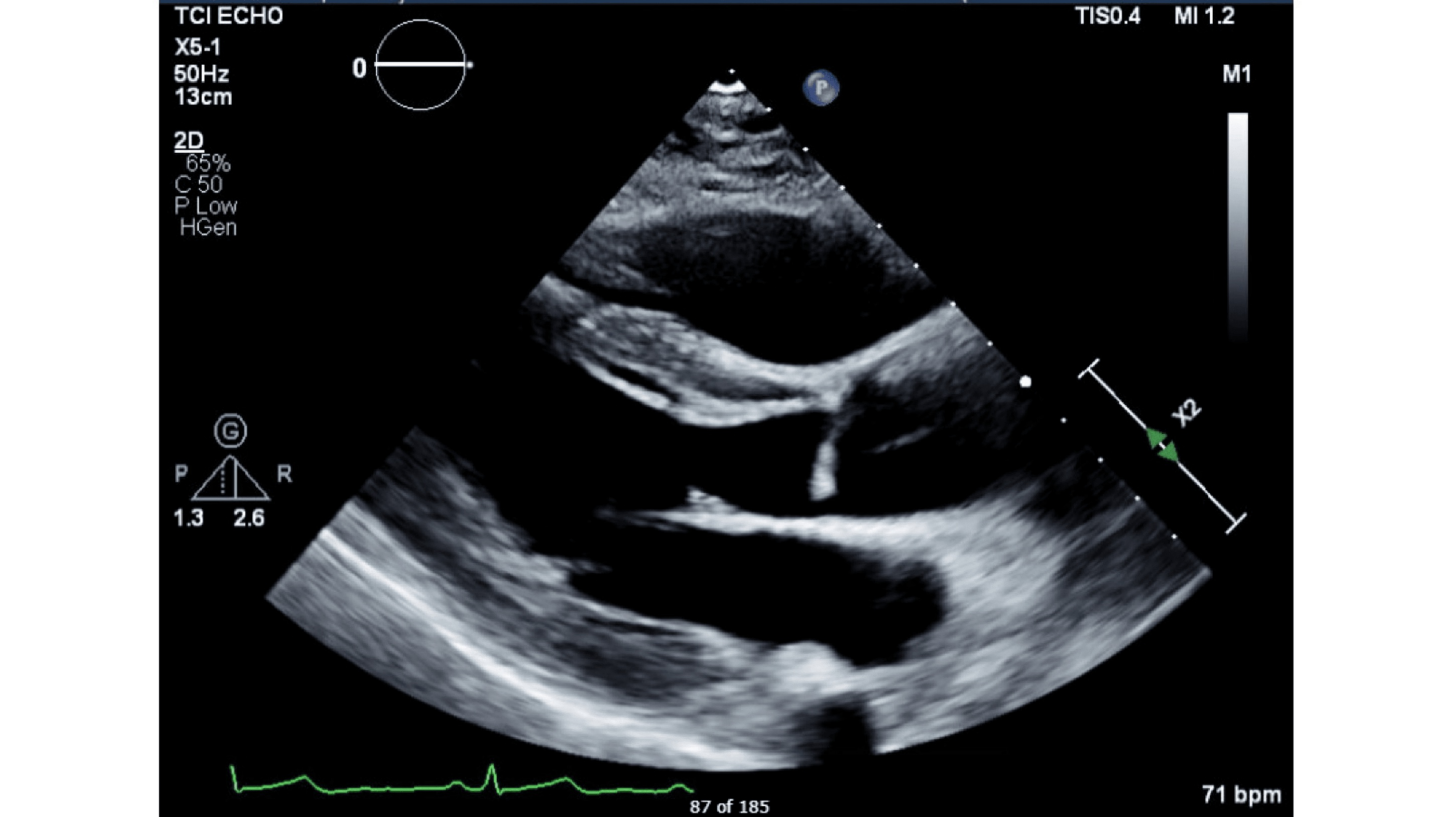
Follow-up transthoracic echocardiogram with parasternal long axis view with normal left ventricular wall motion and ejection fraction 55% to 60%.

Figure 5 shows the parasternal long axis views of follow-up transthoracic echocardiogram at four weeks.

## Discussion

Alcohol is the intoxicating chemical found in beer, cider, wine, and spirits and has been consumed for centuries in many countries around the world [1]. Consumption of small to moderate amounts of alcohol (two to six drinks per week) may be associated with protective cardiac effects, which is observed more with consumption of red and white wine, however consumption of large amounts of alcohol (three to five drinks per day or more) or binge drinking, particularly beer, cider and spirits has been associated with deleterious cardiovascular events and sudden cardiac death [1-3]. There is strong evidence in literature of the relationship of alcohol to arrhythmias based on epidemiological studies, basic science correlates and clinical observation [1-3].

Alcohol has been associated with multiple cardiac arrhythmias including premature atrial and ventricular complexes, atrial fibrillation and atrial flutter, through both direct cardiotoxic effects of alcohol and by inducing electrolyte abnormalities including hypokalemia and hypomagnesemia which results in myocardial instability [1,4]. Atrial fibrillation is the most common cardiac arrhythmia associated with alcohol consumption, even in small to moderate quantities [1,4].

Alcohol is known to cause prolongation of QT interval on the EKG [1,4]. Prolonged QT interval is often worse in patients with alcoholic liver disease. Prolonged QT-interval is known to cause ventricular arrhythmias and sudden cardiac death [1,4]. Patients who are on QT prolonging medications like tricyclic antidepressants, antipsychotics, for example, haloperidol, or the antiemetic ondansetron, have an increased risk of ventricular arrhythmias including Torsades-de-pointes (polymorphic ventricular tachycardia) with alcohol consumption. Patients with congenital long QT-syndrome are also at risk of Torsades-de-pointes. Our patient had no history of congenital long QT-syndrome, and he was not taking any QT-prolonging medications. It is encouraged to discontinue any QT-prolonging medications in patients with a history of alcohol use disorder to reduce this risk. Some suggested mechanisms for increased QT-interval in patients with alcohol use disorder, including alcohol withdrawal, concurrent hypokalemia and hypomagnesemia, altered transmembrane potential and direct damage to the myocardium by alcohol [1].

Many alcoholics often consume other recreational substances, have polysubstance abuse or may be on other QT prolonging medications for anxiety or depression like selective serotonin reuptake inhibitors, which will further potentiate the risk of QT prolongation, cardiac arrhythmias, increase the risk of ventricular tachycardia and sudden cardiac death [1].

Alcohol consumption has been associated with various electrolyte imbalances including hypokalemia, hypomagnesemia and hypophosphatemia, which predispose to myocardial instability, cardiac arrhythmias and increased risk for Torsades-de-pointes and prolonged QT interval [4-7]. One case report describes a patient with a history of severe alcohol use disorder who developed cardiac arrest with severe hypokalemia [7]. Electrolyte imbalances in patients with alcohol use disorder are often difficult to correct and require multiple attempts at repletion due to losses in urine, diarrhea, vomiting, and prolonged deficiency due to malnutrition. Close monitoring of chemistries in these patients is very prudent in management to achieve optimal outcomes [7]. Our patient had hypokalemia of 3.1Meq/L which likely contributed to his ventricular arrhythmia storm and cardiac arrest.

Alcohol consumption over a prolonged period results in altered calcium homeostasis, mitochondrial function, structure and function of contractile proteins which eventually results in impaired myocardial function [1,8]. The cellular and subcellular effects translated to left ventricular dilation and mass increase, as well as left ventricular wall thinning and systolic and diastolic dysfunction [1,8]. Acetaldehyde, which is a metabolite of alcohol, has also been implicated in direct myocardial damage by impairing actin and myosin interaction and causing mitochondrial dysfunction [9].

There is credible evidence in literature of a strong association of alcohol use and arrhythmias, which is highlighted in our patient and in case reports of patients who had similar presentations [10]. Counseling about abstinence should be encouraged to stop the progression of alcohol related cardiac disease. Patients can be given medications like naltrexone and provided social support resources like Alcoholics Anonymous to assist with long-term abstinence. With the end goal of preventing cardiac endpoints like recurrent cardiac arrhythmias, cardiomyopathy, and sudden cardiac death associated with continued alcohol consumption.

Abstinence from alcohol results in improved cardiac outcomes and other systemic benefits. From a cardiac standpoint, abstinence from alcohol has been shown to result in reduced risks of cardiac arrhythmias including atrial fibrillation, atrial flutter, ventricular arrhythmias and reduced risk of sudden cardiac death [1,4]. The risk of hypertension, cardiovascular accident and electrolyte imbalances from hypokalemia, hypomagnesemia and disorder calcium homeostasis is also reduced with alcohol abstinence [1].

Abstinence from alcohol also protects from other systemic deleterious consequences of continued alcohol use like Wernicke's encephalopathy, Korsakoff syndrome, alcoholic liver disease, vitamin deficiencies, malnutrition and other medical conditions associated with continued alcohol use. Abstinence from alcohol also has positive financial and social consequences.

Our patient required a multidisciplinary team. He required aggressive resuscitation and cardiac arrest management by the Emergency medicine physicians and the Intensivist. The Cardiology team managed the alcohol-induced cardiomyopathy, including completing a coronary angiogram, resuming goal-directed medical therapy for heart failure using Carvedilol, Sacubitril-Valsartan and Spironolactone and placed an ICD to prevent future episodes of cardiac arrest. The Psychiatrists and Internists helped with alcohol cessation management. The medical social worker provided resources and follow up for the patient, which helped achieve an optimal outcome.

Patients with alcohol use disorder and in particular those with cardiac risk factors or who have experienced a cardiac event require close management and early out-patient follow up at the time of discharge, with a multidisciplinary approach that includes the primary care providers, behavioral health professionals, cardiologists, medical social worker and other health care providers to achieve optimal long-term outcomes. Abstinence should be encouraged, with resources and social support provided. Patients who have difficulty abstaining from alcohol should be encouraged to attend an alcohol rehabilitation program, which will help them achieve success with quitting. With prompt recognition and management of cardiac arrest due to a ventricular arrhythmia storm as a result of alcohol-induced cardiomyopathy, our patient had a good clinical outcome with recovery of normal cardiac function within 30 days of his initial admission and with continued alcohol abstinence.

Our case highlights the life-threatening cardiotoxic effects of excessive alcohol consumption in a patient who developed alcoholic cardiomyopathy and cardiac arrest, and our approach to management to achieve optimal. Early recognition and management with a multidisciplinary team approach is essential for patient survival and to achieve full recovery.

## Conclusions

Alcohol abuse predisposes to multiple pathophysiologic abnormalities in the heart and has pro-arrhythmic effects through multifactorial mechanisms and electrolyte imbalances. This creates a milieu for producing various cardiac arrhythmias including atrial fibrillation and ventricular arrhythmias, especially in patients with heavy alcohol use or individuals who go on alcohol binge drinking. These could result in cardiac arrest through mechanisms previously described in the discussion, like in our patient and sudden cardiac death. Recommending and encouraging complete abstinence from alcohol and including a multidisciplinary approach, when a correlation is suspected between alcohol use and atrial or ventricular arrhythmias is required to achieve the best prognosis and outcome.
